# Visual preference for social stimuli in individuals with autism or neurodevelopmental disorders: an eye-tracking study

**DOI:** 10.1186/s13229-016-0084-x

**Published:** 2016-04-05

**Authors:** Hayley Crawford, Joanna Moss, Chris Oliver, Natasha Elliott, Giles M. Anderson, Joseph P. McCleery

**Affiliations:** Centre for Research in Psychology, Behaviour and Achievement, Coventry University, James Starley Building (JSG12), Priory Street, CV1 5FB Coventry, UK; Cerebra Centre for Neurodevelopmental Disorders, School of Psychology, University of Birmingham, Birmingham, UK; Institute of Cognitive Neuroscience, University College London, London, UK; School of Psychology, University of Birmingham, Birmingham, UK; School of Psychology, Oxford Brookes University, Oxford, UK; Center for Autism Research, Children’s Hospital of Philadelphia, Philadelphia, PA USA

**Keywords:** Autism spectrum disorder, Fragile X syndrome, Cornelia de Lange syndrome, Rubinstein-Taybi syndrome, Social attention, Eye-tracking

## Abstract

**Background:**

Recent research has identified differences in relative attention to competing social versus non-social video stimuli in individuals with autism spectrum disorder (ASD). Whether attentional allocation is influenced by the potential threat of stimuli has yet to be investigated. This is manipulated in the current study by the extent to which the stimuli are moving towards or moving past the viewer. Furthermore, little is known about whether such differences exist across other neurodevelopmental disorders. This study aims to determine if adolescents with ASD demonstrate differences in attentional allocation to competing pairs of social and non-social video stimuli, where the actor or object either moves towards or moves past the viewer, in comparison to individuals without ASD, and to determine if individuals with three genetic syndromes associated with differing social phenotypes demonstrate differences in attentional allocation to the same stimuli.

**Methods:**

In study 1, adolescents with ASD and control participants were presented with social and non-social video stimuli in two formats (moving towards or moving past the viewer) whilst their eye movements were recorded. This paradigm was then employed with groups of individuals with fragile X, Cornelia de Lange, and Rubinstein-Taybi syndromes who were matched with one another on chronological age, global adaptive behaviour, and verbal adaptive behaviour (study 2).

**Results:**

Adolescents with ASD demonstrated reduced looking-time to social versus non-social videos only when stimuli were moving towards them. Individuals in the three genetic syndrome groups showed similar looking-time but differences in fixation latency for social stimuli moving towards them. Across both studies, we observed within- and between-group differences in attention to social stimuli that were moving towards versus moving past the viewer.

**Conclusions:**

Taken together, these results provide strong evidence to suggest differential visual attention to competing social versus non-social video stimuli in populations with clinically relevant, genetically mediated differences in socio-behavioural phenotypes.

## Background

Eye-tracking technology has been used to differentiate between people with and without autism spectrum disorder (ASD), with relative consistency, using measures of social attention. Furthermore, the extant literature indicates differences in social attention between groups of individuals displaying divergent profiles of social behaviour. For example, reduced attention to social information has been reported in ASD, which is associated with social withdrawal, whereas increased attention to social information has been reported in Williams syndrome, which is associated with hyper-sociability [1–4].

A plethora of research has indicated that people with ASD do not allocate as much attention to social information as typically developing (TD) individuals. For example, studies have reported that people with ASD spend less time than TD individuals viewing people and faces in static pictures of social interactions [4, 5], and further research suggests that reduced face gaze in ASD reflects a lack of interest in social information as it extends to human actors, cartoon images or movies, and clips of naturalistic social scenes [1, 6]. Attention to social stimuli has also been linked to social behaviour [6–8] with reduced social attention being associated with more severe autism symptomatology and consequently more impaired social communicative ability. However, these studies compare looking-time to social and non-social information within a single coherent scene. Therefore, participants in these studies are not required to choose between looking at social information or non-social information as these are both contained within the same stimulus.

When a direct comparison of preference for looking at social versus non-social scenes is used, the findings also reveal that toddlers with ASD do not allocate as much attention to social stimuli as TD toddlers. Pierce and colleagues [9] measured total time spent looking at social video clips (videos of children dancing) compared with videos containing dynamic geometric shapes. Results indicated that toddlers with ASD spent significantly more time fixating on the geometric stimuli than did TD toddlers or toddlers with a developmental delay [9]. Klin and colleagues [10] similarly observed that TD toddlers and toddlers with developmental delays exhibited a visual preference for displays of human biological motion versus inverted displays resembling non-biological motion, whereas toddlers with ASD did not exhibit this preference.

Although some studies contradict this by reporting typical overall looking-times to social versus non-social information in individuals with ASD, more nuanced analyses continue to reveal atypicalities in attention allocation to social information. For example, in a study where a static social scene was presented alongside a static non-social scene, overall looking times did not differ between adolescents with ASD and TD adolescents. However, a preference for social scenes at the first fixation was absent for those with ASD but present for TD individuals [11], indicating reduced attentional prioritisation of social information in ASD.

The nature of social and non-social information may also influence looking patterns in those with ASD. Sasson and Touchstone [12] recently reported no differences between pre-schoolers with ASD and TD pre-schoolers on overall attention allocation to social versus non-social stimuli except for when the non-social stimuli represented common circumscribed interests of children with autism. When non-social stimuli were related to circumscribed interests, participants with ASD allocated less attention to social stimuli than TD controls. In addition, the ecological validity of social stimuli has been reported to influence attentional abnormalities in ASD. Specifically, videos of social interaction produced more sensitive group differences than videos of individual stimuli or static stimuli [13].

Although visual attention to social versus non-social information has been explored extensively in individuals with ASD, to date, studies using preferential looking paradigms to examine looking patterns to directly competing, dynamic, social, and non-social stimuli, such as those reported by Pierce and colleagues and Klin and colleagues [9, 10], have only used stimuli that are facing the participant. It is important to look at the factors that may influence typical and atypical social attention in individuals with ASD and other neurodevelopmental disorders. One possibility is that social information may be more threatening to individuals with ASD, which has been associated with heightened social anxiety (see [14] for a review) and social impairment. The current study aims to explore this further by presenting social and non-social stimuli, where the actor or object is either moving towards or moving past participants, to individuals with different neurodevelopmental disorders that are each differentially associated with social anxiety and social impairment. It has been proposed that biological motion that is facing towards the viewer is potentially more threatening than stimuli that are oriented away from the viewer. This ‘facing-the-viewer’ bias has also been associated with a heightened state of physiological arousal [15]. Therefore, the ‘moving towards’ stimuli presented in the current study are proposed to be more threatening than the ‘moving past’ stimuli. Further, individuals with anxiety without a neurodevelopmental disorder have been reported to show faster orienting to threatening stimuli, but not pleasant stimuli, when compared to non-anxious individuals (see [16] for a review). Although the current study does not directly measure anxiety in individuals with ASD, it is possible to postulate the extent to which social anxiety or social indifference governs atypical social attention in ASD. For example, social anxiety may subserve a pattern of results whereby participants demonstrate reduced looking to social stimuli only when it is moving towards them whereas reduced looking to both sets of social stimuli would more likely be governed by social indifference.

Although the effect that this particular stimulus feature has on attentional allocation in ASD has not yet been investigated in depth, some study results have suggested that differences may emerge when this subtle factor is manipulated. For example, Chawarska and colleagues showed that when dyadic communication cues were introduced in a video of an actress making a sandwich, toddlers with ASD spent less time looking at scenes, and the actor in scenes, involving direct communication when the toys were moving in the background, than TD toddlers and toddlers with a developmental disability but no ASD. This suggests that the toddlers with ASD do not show a general deficit in attending to people but, rather, that reduced attention becomes apparent only in the presence of direct communication bids [17]. Similarly, increased activation in a number of brain regions has been reported when TD participants have observed a male walking towards them with direct gaze compared with averted gaze, which has not been replicated in ASD [18]. The manipulation of 'directed towards' used in these studies is perhaps the most similar manipulation that has been used in the existing literature to date, to the current 'moving towards' versus 'moving past' manipulation.  These studies are discussed above as they provide a starting point for guiding hypotheses. However, the direction of stimuli was not assessed in these studies so we cannot assume that this was driving these results. Furthermore, ‘moving towards’ versus ‘moving past’ was used in the present study in order to potentially increase the contrast between degrees of threat.

Although social attention abnormalities have also been demonstrated in ASD using stimuli that are not facing participants [6], incorporating this subtle experimental manipulation into a relatively simple preferential looking paradigm with directly competing social and non-social stimuli allows further delineation of social information processing in this group in a way that can break down the features of the stimuli that may result in abnormal attention patterns. Delineating this potential relationship is more achievable using a preferential looking paradigm as opposed to when facing and non-facing social and non-social stimuli are incorporated within a singular scene or video, which is largely used in the existing literature.

In the current studies, we use a preferential looking paradigm to explore social attention to dynamic social and non-social stimuli that are either moving towards or moving past the viewer in adolescents with ASD versus individuals with special education needs (SEN) without ASD (study 1). As a relationship between social behaviour and social information processing has previously been documented [1–4, 6–8], we aim to further examine the value and validity of this paradigm to index variability in social-behavioural phenotypes. To do this, we employ this same paradigm to examine social attention in individuals with three different genetic syndromes associated with unique social profiles: fragile X (FXS), Cornelia de Lange (CdLS), and Rubinstein-Taybi syndromes (RTS; study 2).

Previous literature, such as that reported above, has focussed on atypicalities in social information processing to help explain some of the social interaction difficulties observed in children and adults with ASD. Limited research has been conducted to further understand social information processing skills in children and adults with neurodevelopmental disorders, other than ASD that are also associated with social interaction difficulties. The three genetic syndromes studied here are associated with varied profiles of social behaviour, some aspects of which are comparable across the syndromes whilst other aspects are subtly different. One aim of this study is to use implicit measures, which reduce performance demand, to compare and contrast social information processing in FXS, CdLS, and RTS. Understanding social cognition may have important implications for further understanding the socio-behavioural impairments associated with these syndrome groups. However, as FXS, CdLS, and RTS are associated with intellectual disability, using measures that are typically used in mainstream social cognition literature may influence results and indicate impairments in social cognition that are more likely a result of task demands.

Across a number of studies using eye-tracking methodology, Riby and colleagues have consistently reported a link between visual processing of social information using implicit measures and socio-behavioural characteristics. Specifically, individuals with Williams syndrome, which is associated with hyper-sociability, have been shown to spend more time looking at faces, and the eye region of faces, than TD participants [1, 2, 4]. On the other hand, the same series of studies has highlighted that individuals with ASD, which is associated with social withdrawal, spend less time looking at faces and eyes than TD participants. In addition, individuals with Williams syndrome have shown stronger emotional expression processing skills than individuals with ASD [19] and a greater ability to interpret cues from eye gaze [20]. These studies point to lower levels of interest in social information in individuals exhibiting social withdrawal and heightened interest in social information in individuals exhibiting hyper-sociability.

FXS is the most common cause of inherited intellectual disability [21], affecting approximately 1 in 4000 males and 1 in 8000 females [22]. FXS has been associated with social anxiety, shyness, and eye gaze aversion [23, 24]. However, it has been suggested that these avoidant behaviours occur primarily during initial interactions, giving way to increasing social approach behaviours over time [25, 26]. As an X-linked disorder, females display fewer cognitive and social impairments [27]. CdLS is a genetic disorder affecting approximately 1 in 40,000 live births [28] and is associated with intellectual disability, selective mutism, social anxiety, and shyness [29–32]. RTS is also a genetic syndrome associated with intellectual disability affecting approximately one in 100,000–125,000 live births [33]. In contrast to FXS and CdLS, research generally suggests that individuals with RTS are sociable, with higher levels of social interest and social contact compared with a matched contrast group [34].

Due to the comparative rarity of FXS, CdLS, and RTS to ASD, the literature regarding social information processing is more limited in these genetic syndrome groups. One study that has investigated social information processing in FXS measured looking patterns to photographic scenes that incorporated social stimuli but manipulated the location of the social stimuli within the scene, thereby allowing comparison of attention allocation to social information when non-social information is also available [35]. No differences in the amount of time spent looking at social information were reported between those with FXS and controls matched on chronological and mental age. However, participants with FXS were faster than TD participants to look away, indicating active social avoidance. These results suggest that more nuanced analyses including speed of gaze aversion may highlight subtle differences. Out of the 14 participants with FXS in the study conducted by Williams et al. [35], 12 were female. Due to documented gender differences in FXS, it cannot be determined whether the same results would extend to males with FXS. Other studies utilising eye-tracking technology to investigate social information processing in FXS have focussed on looking patterns to faces. As faces are social in nature, visual preference for social or non-social information cannot be gleaned from these studies. However, they do provide evidence that social processing may be impaired in this syndrome group. These studies have been conducted primarily to investigate looking patterns to the eye region of facial stimuli and report reduced time spent looking at the eyes in participants with FXS compared to TD participants [36–38] and compared to individuals with ASD [39].

### Hypotheses

It was hypothesised that individuals with ASD would exhibit reduced looking to social versus non-social stimuli that is moving towards the viewer, when compared with a group of individuals with SEN who were matched for chronological age (CA) and verbal abilities but did not have ASD. This hypothesis was based directly upon previous research in which stimuli were people facing towards the camera [9, 10]. This study also allows for examination of whether or not the relative reduced looking at social versus non-social stimuli is present for stimuli that is moving past the viewer.

Previous literature indicates differences in social processing that map onto social behaviours [1, 4]. Due to subtle differences in the documented socio-behavioural characteristics of the genetic syndrome groups of focus in this study, between-group differences in visual attention for social videos were hypothesised. Specifically, due to reports of sociability and social interest in RTS, it was predicted that participants with RTS would direct more visual attention towards social versus non-social stimuli, whereas individuals with FXS and CdLS would not exhibit this visual preference, in line with the reported social anxiety and shyness observed in these groups. These hypotheses were based upon the documented socio-behavioural phenotypes of the syndrome groups. However, the absence of previous literature on social versus non-social preference in the syndrome groups precludes us from making strong specific predictions (for visual scanning of social stimuli only, see [36–40] for FXS and [41] for CdLS and RTS). Due to the documented socio-behavioural profiles of both FXS and CdLS indicating social anxiety, a parental-report measure of this behaviour was included in the present study in an effort to investigate the potential relationship between social anxiety and visual attention towards social stimuli. Whilst it would have been interesting to investigate this potential relationship in the ASD group, limited access to parents when testing adolescents with ASD in a school setting rendered this unfeasible. Furthermore, because FXS, CdLS, and RTS are associated with intellectual disability, it was not possible to compare the ASD or SEN participants in the current study. Specifically, due to the wide range of chronological ages and ability levels in our participants with FXS, CdLS, and RTS, the Vineland Adaptive Behavior Scale (VABS)-II [42] was used in place of an intellectual quotient (IQ) measure. However, it was possible to obtain verbal IQ data on participants with ASD and SEN. The group comparisons are therefore split into two studies. The first study reports data from participants with ASD and SEN who are matched on chronological age, gender, and verbal IQ. The second study reports data from participants with FXS, CdLS, and RTS who are matched on chronological age and adaptive behaviour.

## Study 1

### Methods

#### Participants

Sixteen adolescents with ASD and 16 adolescents with SEN but no diagnosis of a neurodevelopmental disorder were included in study 1. All participants were recruited from a secondary school local to the research base and had normal or corrected to normal vision. An educational psychologist had previously diagnosed all 16 participants in the ASD group and ruled out a diagnosis of ASD in all 16 adolescents in the SEN group. The Autism Diagnostic Observation Schedule (ADOS) [43] was administered by a research-trained examiner to confirm the presence or absence of a diagnosis in participants in the ASD and SEN groups, respectively. The verbal similarities and word definitions portions of the school-age British Ability Scales—second edition [44] were administered to all participants in order to provide standardised information on verbal abilities. Participant characteristics are presented in Table 1.

**Table 1:** Participant characteristics and comparison statistic for adolescents with autism spectrum disorder (ASD) and special educational needs (SEN)

	ASD	SEN	*p* value
	(*n* = 16)	(*n* = 16)	
CA mean (SD)	13.33 (.62)	13.06 (.90)	.323
Gender percentage female	6.25	6.25	1.00
Verbal ability standard scores (SD)	71.94 (18.55)	77.75 (15.17)	.340
ADOS mean score (SD)	12.67 (3.958)	2.13 (2.094)	< .001

#### Ethics, consent, and permissions

Parents of participants were sent information about the study and given the opportunity to opt their child out of participation. Additionally, all participants provided fully informed written consent prior to participation. This consent process was in accordance with an ethical protocol that was approved by the Science, Technology, Engineering, and Mathematics Ethical Review Committee at the University of Birmingham.

#### Apparatus

An EyeLink 1000 Tower Mount system was used to measure participant’s dwell time and eye movements. It has a temporal resolution of 2 ms (500 Hz), spatial accuracy of 0.5°–1° visual angle, and a spatial resolution of 0.01°.

#### Stimuli

**Fig. 1:**
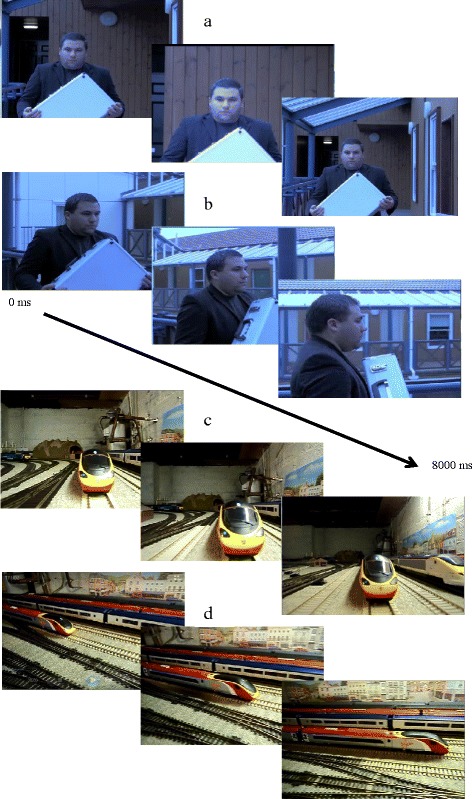
An example of the dynamic stimuli presented during the social ‘moving towards’ (**a**), social ‘moving past (**b**), non-social ‘moving towards (**c**), and non-social ‘moving past (**d**) conditions. Written informed consent for publication of their image was obtained from the actor in (**a**) and (**b**)

During each trial, participants were presented with two videos side by side for 8000 ms. The videos were either social, where an actor was the focus of the video, or non-social, where an object was the focus of the video. Both videos were either ‘moving towards’ or ‘moving past’. In the ‘moving towards’ videos, the person or object moved towards, or conducted an action (e.g., blowing bubbles) towards, the viewer. In the ‘moving past’ videos, the person or object moved past or conducted an action past the camera in a perpendicular fashion. Figure 1 shows an example of stimuli at three time points (between 0 and 8000 ms) in each of the conditions: the social ‘moving towards’ condition (Fig. 1a), the social ‘moving past’ condition (Fig. 1b), the non-social ‘moving towards’ condition (Fig. 1c), and the non-social ‘moving past’ condition (Fig. 1d). There were 28 trials in total, half of which contained one social video moving towards the viewer and one non-social video moving towards the viewer, whilst the other half contained one social video moving past the viewer and one non-social video moving past the viewer. Trials were counterbalanced so that the social and non-social videos were presented an equal number of times on the left and right side of the screen. Examples of social videos include a person skipping, a person blowing bubbles, and a person walking whilst talking on the phone. Examples of non-social videos include a train, an aeroplane, and a ball bouncing down steps. Actors in all of the videos wore plain black clothing and displayed a straight-ahead gaze and neutral facial expression. Each video subtended an average of 9.15 × 13.79° of visual angle and was displayed on a white background. The videos were positioned side by side, separated by a gap of 1.25° of visual angle.

#### Procedure

All participants were tested at their school and were seated approximately 50 cm from the computer screen displaying the stimuli. A five-point calibration was performed prior to the experiment in which participants looked at an animated blue dolphin that changed position around the screen. Following calibration, participants were presented with 28 trials. In between each trial, the animated dolphin, which served as a central fixation point, was displayed for 1000 ms, except for prior to every fifth trial when a single-point calibration drift-correction was made. Participants were told to look wherever they wished on the computer screen whilst the videos were presented but to look at the dolphin in between trials.

#### Data analysis

The current study uses measures of overall dwell time to social versus non-social stimuli and time taken to orient to social versus non-social stimuli, as well as incorporating the manipulation of the direction of the stimuli. Fixations were assessed as occurring when eye movement did not exceed a velocity threshold of 30°/s, an acceleration threshold of 8000°/s^2^, or a motion threshold of 0.1°, and the pupil was not missing for three or more samples in a sequence. To determine whether participant groups differed in the amount of time spent looking at social relative to non-social videos, the mean proportion of dwell time to stimuli moving towards (Equation 1) and stimuli moving past (Equation 2) the viewer was calculated for each participant. This indicates what proportion, out of the total time spent looking at both videos on the screen, was spent viewing the social stimuli.

Equation 1. Dwell time formula for stimuli moving towards the viewer.

1 $$ \frac{\mathrm{Mean}\ \%\ \mathrm{o}\mathrm{f}\ \mathrm{dwell}\ \mathrm{time}\ \mathrm{o}\mathrm{n}\ \mathrm{social}\ `\mathrm{moving}\ \mathrm{towards}'\ \mathrm{videos}}{\left(\mathrm{Mean}\ \%\ \mathrm{o}\mathrm{f}\ \mathrm{dwell}\ \mathrm{time}\ \mathrm{o}\mathrm{n}\ \mathrm{social}\ `\mathrm{moving}\ \mathrm{towards}'\ \mathrm{videos} + \mathrm{mean}\ \%\ \mathrm{o}\mathrm{f}\ \mathrm{dwell}\ \mathrm{time}\ \mathrm{o}\mathrm{n}\ \mathrm{n}\mathrm{o}\mathrm{n}\hbox{-} \mathrm{social}\ `\mathrm{moving}\ \mathrm{towards}'\ \mathrm{videos}\right)} $$

Equation 2. Dwell time formula for stimuli moving past the viewer.

2 $$ \frac{\mathrm{Mean}\ \%\ \mathrm{o}\mathrm{f}\ \mathrm{dwell}\ \mathrm{time}\ \mathrm{o}\mathrm{n}\ \mathrm{social}\ `\mathrm{moving}\ \mathrm{past}'\ \mathrm{videos}}{\left(\mathrm{Mean}\ \%\ \mathrm{o}\mathrm{f}\ \mathrm{dwell}\ \mathrm{time}\ \mathrm{o}\mathrm{n}\ \mathrm{social}\ `\mathrm{moving}\ \mathrm{past}'\ \mathrm{videos} + \mathrm{mean}\ \%\ \mathrm{o}\mathrm{f}\ \mathrm{dwell}\ \mathrm{time}\ \mathrm{o}\mathrm{n}\ \mathrm{n}\mathrm{o}\mathrm{n}\hbox{-} \mathrm{social}\ `\mathrm{moving}\ \mathrm{past}'\ \mathrm{videos}\right)} $$

To determine whether participant groups differed in their speed to fixate to social relative to non-social videos, the mean ratio of the latency of first fixations to social versus non-social videos was calculated for each participant for stimuli moving towards (Equation 3) and stimuli moving past (Equation 4) the viewer. A ratio above 1 reflects quicker fixation to social stimuli, so, for example, a ratio of 3 indicates that participants fixated to social stimuli three times faster than non-social stimuli.

Equation 3. First fixation latencies formula for stimuli moving towards the viewer.

3 $$ \frac{\mathrm{Mean}\ \mathrm{t}\mathrm{ime}\ \mathrm{t}\mathrm{aken}\ \mathrm{t}\mathrm{o}\ \mathrm{fixate}\ \mathrm{o}\mathrm{n}\ \mathrm{n}\mathrm{o}\mathrm{n}\hbox{-} \mathrm{social}\ `\mathrm{moving}\ \mathrm{t}\mathrm{o}\mathrm{wards}'\ \mathrm{videos}}{\mathrm{Mean}\ \mathrm{t}\mathrm{ime}\ \mathrm{t}\mathrm{aken}\ \mathrm{t}\mathrm{o}\ \mathrm{fixate}\ \mathrm{o}\mathrm{n}\ \mathrm{social}\ `\mathrm{moving}\ \mathrm{t}\mathrm{o}\mathrm{wards}'\ \mathrm{videos}} $$

Equation 4. First fixation latencies formula for stimuli moving past the viewer.

4 $$ \frac{\mathrm{Mean}\ \mathrm{t}\mathrm{ime}\ \mathrm{t}\mathrm{aken}\ \mathrm{t}\mathrm{o}\ \mathrm{fixate}\ \mathrm{o}\mathrm{n}\ \mathrm{n}\mathrm{o}\mathrm{n}\hbox{-} \mathrm{social}\ `\mathrm{moving}\ \mathrm{past}'\ \mathrm{videos}}{\mathrm{Mean}\ \mathrm{t}\mathrm{ime}\ \mathrm{t}\mathrm{aken}\ \mathrm{t}\mathrm{o}\ \mathrm{fixate}\ \mathrm{o}\mathrm{n}\ \mathrm{social}\ `\mathrm{moving}\ \mathrm{past}'\ \mathrm{videos}} $$

These ratios were subjected to a logarithmic (Lg^10^) transformation in order to meet criteria for normal distribution. Data from one participant with ASD, one participant with SEN, one participant with RTS, and two participants with FXS were excluded from parametric analyses, as they could not be transformed to normality due to ratios below zero. Due to the potential bias this creates in the data, we also confirmed the findings using non-parametric tests, performed with the original ratios that were not normally distributed.

## Results

### Dwell time

**Fig. 2:**
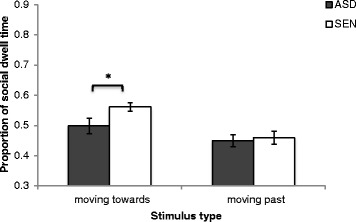
The mean (±1 SE) proportion of social dwell time on 'moving towards' and 'moving past' videos for adolescents with autism spectrum disorder (*ASD*) and adolescents with special educational needs (*SEN*)

On average, participants with ASD and SEN spent 92 and 86 % of trial time looking at the videos, respectively, indicating good levels of task engagement. Figure 2 depicts the proportion of dwell time on social versus non-social stimuli in the ‘moving towards’ and ‘moving past’ conditions. A 2 × 2 mixed ANOVA was conducted where direction (moving towards/moving past) was the within subjects factor and participant group (ASD, SEN) was the between subjects factor. A main effect of direction was revealed (*F* (1, 30) = 17.029, *p* < .001). Neither a main effect of participant group (*F* (1, 30) = 2.657, *p* = .114) nor a significant interaction was observed (*F* (1, 30) = 2.112, *p* = .156). As previous studies have used stimuli facing the viewer and have consistently observed effects of participants with ASD looking less to social stimuli than control participants [9, 10], we employ two hypothesis-driven a priori independent *t* tests in order to determine whether or not these effects replicate in the current data. These revealed that adolescents with SEN demonstrated a significantly higher proportion of social versus non-social looking at stimuli moving towards the viewer compared to adolescents with ASD (*t* (30) = 2.183, *p* = .037). This difference was not observed for stimuli moving past the viewer (*t* (30) = .346, *p* = .732).

In order to ensure that the observed effects were not driven by circumscribed interests, such as interest in vehicles in the ASD group, image-wise analyses were conducted whereby a dwell time of 2× standard deviations above or below the group mean dwell time was considered an outlier. These analyses revealed that none of the images used in the present study yielded dwell times that were deemed outliers for the ASD group.

### First fixation latencies

**Fig. 3:**
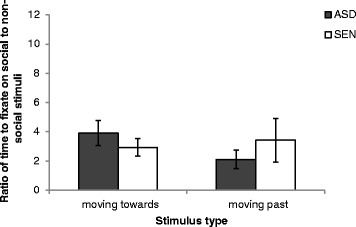
The mean (±1 SE) ratio of first fixation latencies on social to non-social stimuli for adolescents with autism spectrum disorder (ASD) and adolescents with special educational needs (SEN)

In the following analyses, a larger ratio indicates quicker fixation to social versus non-social stimuli. Figure 3 depicts the first fixation latencies for social versus non-social stimuli in the ‘moving towards’ and ‘moving past’ conditions. A 2 (moving towards/moving past) × 2 (ASD/SEN) mixed ANOVA was conducted. No main effects or a significant interaction were observed (moving towards/moving past: *F* (1, 28) = 3.029, *p* = .093; ASD/SEN: *F* (1, 28) = .149, *p* = .702; interaction: *F* (1, 28) = 1.091, *p* = .305).

## Study 2

### Methods

Except where mentioned, the methods used in study 2 were identical to those used in study 1.

#### Participants

Fifteen individuals with FXS, 14 individuals with CdLS, and 19 individuals with RTS were included in study 2. Participant characteristics are presented in Table 2. Due to documented gender differences, all participants with FXS were male. Participants were either recruited through the participant database of the Cerebra Centre for Neurodevelopmental Disorders, the Cornelia de Lange Foundation UK and Ireland, or the Rubinstein-Taybi Syndrome UK Support Group. All participants had previously received a diagnosis by a paediatrician or clinical geneticist and had normal or corrected to normal vision. Participant groups were matched for CA, global adaptive behaviour, and verbal adaptive behaviour as measured by the VABS [42].

**Table 2:** Participant characteristics and comparison statistic for children and adults with fragile X (FXS), Cornelia de Lange (CdLS), and Rubinstein-Taybi (RTS) syndromes

	FXS	CdLS	RTS	*p* value
	(*n* = 15)	(*n* = 14)	(*n* = 19)	
CA mean (SD)	24.21 (8.61)	18.21 (5.59)	20.94 (11.94)	.303^a^
Gender percentage female	0	57.14	73.68	<.001^b^
Adaptive behavior composite (SD)	47.80 (14.64)	51.29 (17.42)	47.89 (15.95)	.798
Adaptive behaviour—communication (SD)	39.40 (17.56)	48.79 (21.94)	47.94 (17.70)	.332
ADOS mean score (SD)	9.6 (5.25)	NA	NA	NA
Participants meeting ASD cut-off	11	NA	NA	
Participants meeting autism cut-off	5	NA	NA	

^a^Participants were also matched on chronological age when compared separately (FXS versus CdLS: *t* (27) = 1.774, *p* = .087; FXS versus RTS: *t* (32) = .891, *p* = .380; CdLS versus RTS: *t* (31) = −.703, *p* = .487)

^b^Participants with CdLS and RTS were matched on gender (*χ* ^2^ (1) = .992, *p* = .319)

#### Ethics, consent, and permissions

Participants aged 16 years and over, and parents of participants aged under 16 years, provided fully informed written consent to participate in the study. If necessary, participants aged 16 years and over were provided with a symbol sheet to explain the experimental procedure using pictures and short sentences. Participants were also given a ‘stop’ card, which they could hold up if they wanted a break or to stop the experiment, although this was not used by any participant. This was in accordance with an ethical protocol that was approved by the Science, Technology, Engineering, and Mathematics Ethical Review Committee at the University of Birmingham.

#### Procedure

Participants were tested individually in a quiet, dimly lit room either at the University of Birmingham (FXS = 15; CdLS = 2) or at a syndrome support group family meeting (CdLS = 12; RTS = 19). All participants were seated approximately 60 cm from the computer screen displaying the stimuli. Parents/primary caregivers completed the VABS [42], the Social Communication Questionnaire (SCQ) [45], and the parent version of the Spence Children’s Anxiety Scale (SCAS-P) [46]. A researcher who was trained in ADOS administration at research-reliable level administered the ADOS [43] to all participants with FXS.

## Results

### Dwell time

**Fig. 4:**
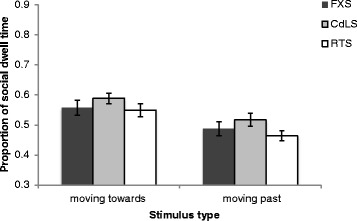
The mean (±1 SE) proportion of social dwell time on 'moving towards' and 'moving past' videos for participants with fragile X (FXS), Cornelia de Lange (CdLS), and Rubinstein-Taybi (RTS) syndromes

On average, participants with FXS, CdLS, and RTS spent 83, 89, and 92 % of trial time looking at the videos, respectively, indicating good levels of task engagement. Figure 4 depicts the proportion of social versus non-social dwell time in the ‘moving towards’ and ‘moving past’ conditions. A 2 (moving towards/moving past) × 3 (FXS/CdLS/RTS) mixed ANOVA revealed a main effect of direction; participants evidenced a higher proportion of social versus non-social looking for stimuli moving towards versus moving past the viewer (*F* (1, 45) = 45.886, *p* < .001, *η*^2^ = .505). There was no significant main effect of group or a significant interaction. As all participants with FXS were male, the three groups were not matched on gender. Therefore, these analyses were re-conducted with the CdLS and RTS groups only, who were matched on gender, to ensure that this did not affect results. These analyses revealed that the main effect of direction, and lack of significant findings for participant group and the interaction, remained the same when only the CdLS and RTS groups were compared.

### First fixation latencies

**Fig. 5:**
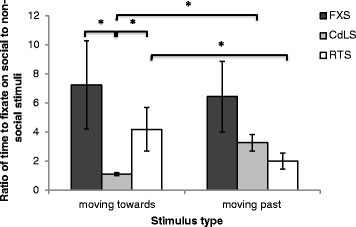
The mean (±1 SE) ratio of first fixation latencies on social to non-social stimuli for participants with fragile X (FXS), Cornelia de Lange (CdLS), and Rubinstein-Taybi (RTS) syndromes

Figure 5 depicts the first fixation latencies to social versus non-social stimuli in the ‘moving towards’ versus ‘moving past’ conditions. A 2 (moving towards/moving past) × 3 (FXS/CdLS/RTS) mixed ANOVA revealed a main effect of participant group (*F* (2, 42) = 3.566, *p* = .037, *η*^2^ = .145) and a significant interaction (*F* (2, 42) = 4.821, *p* = .013, *η*^2^ = .187), indicating differential impact of the direction of stimuli (moving towards/moving past) on the time taken to fixate on social relative to non-social stimuli across the participant groups. Bonferroni corrected post hoc tests indicated slower fixation to social relative to non-social stimuli that were moving towards the viewer in the CdLS group compared to the FXS (*p* < .001) and RTS (*p* = .005) groups. This between-group difference for ‘moving towards’ stimuli was confirmed with a Kruskal-Wallis test (*χ*^2^ (2) = 21.070, *p* < .001) and follow-up Mann-Whitney *U* tests (FXS versus CdLS: *U* = 12.00, *p* < .001; FXS versus RTS: *U* = 92.00, *p* = .080; CdLS versus RTS: *U* = 34.00, *p* < .001). These analyses include a direct comparison between participants with CdLS and RTS and, therefore, show that the results are unlikely to be driven by gender differences as gender is matched across these two groups. Paired samples *t* tests further revealed that participants with CdLS fixated to social relative to non-social stimuli slower when stimuli were moving towards versus moving past the viewer (*t*(13) = −4.415, *p* = .001; confirmed with Wilcoxon Signed Ranks Test, *Z* = −3.107, *p* = .002), whereas participants with RTS demonstrated the opposite pattern (*t*(17) = 2.247, *p* = .038; confirmed with Wilcoxon Signed Ranks Test, *Z* = −2.213, *p* = .027), and participants with FXS showed no difference between ‘moving towards’ and ‘moving past’ ratios (*p* > .051; confirmed with Wilcoxon Signed Ranks Test, *Z* = −.682, *p* = .496).

### Association between participant characteristics and social preference

Spearman correlations revealed a significant positive association between the proportion of social dwell time on stimuli moving towards the viewer and SCAS-P total anxiety score (r_s_ (12) = .667, *p* = .009) and social phobia subscale score (r_s_ (12) = .548, *p* = .043) for the FXS group only. There were no significant relationships between autism symptomatology, as measured by the SCQ, and dwell time on stimuli moving towards or moving past the viewer for any participant group (*p* > .05). No correlations were revealed for SCAS-P scores and first fixation latencies for any participant group (*p* > .05). However, a moderate positive relationship was revealed between autism symptomatology and first fixation latencies to stimuli moving towards the viewer for the RTS group only (r_s_ (16) = .664 (*p* = .003).

## Discussion

In the current studies, we used eye-tracking measures in conjunction with competing social and non-social videos, under ‘moving towards’ and ‘moving past’ conditions. The aim of this work was to determine whether or not indices of visual preferences or visual salience distinguish among individuals with and without ASD and whether this measure is sensitive to differences between other groups of individuals with differing socio-behavioural phenotypes as a result of rare genetically mediated syndromes (FXS, CdLS, and RTS).

Consistent with existing literature, adolescents with SEN evidenced a higher proportion of dwell time for dynamic social versus non-social stimuli that were moving towards them, compared with adolescents with ASD. Extending the previous literature, the present study reported that this difference was not apparent for stimuli that were not facing participants. In study 2, analyses indicated that participants with CdLS took longer to fixate to social videos moving towards the viewer than did participants with FXS or RTS, whereas participants with FXS and RTS did not differ on this index. Together, these results suggest that eye-tracking measures of attentional maintenance and prioritisation to dynamic, social and non-social stimuli that are moving towards viewers can differentiate between groups of typical versus atypical social development (study 1), as well as between groups with subtly different socio-behavioural profiles (study 2).

Interestingly, although the groups across the two studies were not directly compared, a visual comparison of Figs. 2 and 4 show that participants with ASD evidenced a lower proportion of dwell time for ‘moving towards’ social versus non-social stimuli than did participants with FXS, CdLS, and RTS, whose looking times more similarly reflected participants in the SEN group. In the ‘moving past’ condition, it appears that participants with FXS, CdLS, and RTS showed a slightly higher proportion of dwell time for social versus non-social stimuli than did participants with ASD and SEN. Furthermore, a visual comparison of Figs. 3 and 5 indicate that participants with FXS fixated quicker, and participants with CdLS fixated slower, on social ‘moving towards’ stimuli than any of the five participant groups. Participants with FXS also fixated quicker on social ‘moving past’ stimuli than any other participant group. It was not possible to statistically compare the patterns of results across the groups in these two separate studies because they were not matched on a number of important participant characteristics. However, the patterns observed suggest that future studies should aim to recruit and test matching participant groups in order to explore this further.

The results from study 1 support and extend previous research that has observed that people with ASD do not allocate as much attention to social information as do TD individuals when social stimuli are presented alongside geometric images and point light displays of non-biological motion [9, 10]. This highlights the consistency of findings across ages in this population, from children through to adolescents, and suggests that reduced attention to social information is also apparent when social stimuli are presented alongside more naturalistic non-social stimuli consisting of objects commonly seen in everyday life. This study further highlights the important role of stimulus manipulation and, in particular, the use of stimuli that are facing the participant, when distinguishing differences in attentional allocation between groups with and without social impairment. These data pose interesting questions for the role of anxiety in attentional allocation to social stimuli in adolescents with ASD. It was postulated that social anxiety, rather than social indifference, would more likely govern visual attention to social stimuli if reduced looking was found only in the ‘moving towards’ condition. As this was the case, it is important to further explore the relationship between anxiety and social attention in ASD.

Whilst overall looking time to social versus non-social stimuli did not differ between genetic syndrome groups, participants with CdLS took longer to fixate to social stimuli moving towards the viewer than the other groups. This suggests that attentional prioritisation of socially salient stimuli is reduced in individuals with CdLS compared to those with FXS and RTS. This pattern of results in CdLS may be related to social anxiety, or a reduced ability to interact, both of which have previously been highlighted in this group [47, 48]. However, these characteristics have also been associated with FXS, indicating potentially differential relationships between social behaviour and social attention for those with CdLS versus FXS, despite perceptions in the research community of similar behavioural profiles based on limited data.

A positive association between social dwell time to stimuli moving towards the viewer and anxiety was revealed in the FXS group only. This may reflect hypervigilance and heightened attention towards threatening stimuli. In the present study, it may be the case that approaching social stimuli are perceived as more threatening than non-approaching social stimuli. However, social anxiety has also been reported in individuals with CdLS [47], but measures of attentional priority for social information in this study indicate differences between these two groups on this measure. This seems to provide some degree of support for the possibility proposed above that attentional priority for social information may have a differential association with social behaviour across individuals with FXS and CdLS. Finally, the results for individuals with RTS are generally consistent with some of the existing, albeit limited, reports of the social phenotype of this group. For example, individuals with RTS have been reported to display social interest, intact social skills relative to their intellectual ability, and the ability to initiate and maintain social contacts [34, 49, 50]. Specifically, this group exhibited increased attentional maintenance of attention towards socially salient (moving towards) stimuli compared to less salient (moving past) social stimuli and increased attentional prioritisation to social stimuli compared to those with CdLS. Notably, our previous studies of social attention in CdLS and RTS revealed no differences in eye- and mouth-looking [41], highlighting the importance of specific manipulations to social attention paradigms.

The current study focuses on drawing comparisons in looking patterns to social versus non-social stimuli, whilst investigating the relative effect that stimulus direction has on visual preference. To our knowledge, this is the first study to systematically manipulate the extent to which stimuli moves towards versus past the viewer. An interesting avenue for future research would be to study the effects of stimulus direction independently by presenting pairs of social and non-social stimuli separately, where the actor or object in one video moves towards the viewer and the actor or object in the other video moves past the viewer. Study 1 documents reliable eye-tracking data from 16 participants with ASD and 16 control participants. Although this sample size is acceptable, obtaining a larger sample size may have resulted in stronger effects. Study 2 produced and examined reliable eye-tracking data from one of the largest samples of males with FXS and participants with CdLS and RTS, three rare genetic syndromes. This is the first study to compare participants with these three genetic syndromes, each associated with subtly different social profiles, on a measure of social attention.

## Conclusions

In summary, the results of the two studies presented here suggest that eye-tracking measures of social versus non-social video stimulus preferences and prioritisation index differences between groups of individuals with differing social profiles. Specifically, those with versus without ASD exhibited differences on a relatively coarse measure of overall dwell time to social versus non-social videos, whilst the nuanced measure of time taken to initially orient to social and non-social stimuli highlighted differences between groups exhibiting more subtle differences in their social presentation. Critically, the differences observed between the groups are each consistent with previously documented differences in their respective behaviourally measured social phenotypes. The current findings, therefore, provide further support for the potential of relatively simple eye-tracking measures of visual attention for social versus non-social stimuli to index differences across populations according to their socio-behavioural profiles.

### Ethics, consent, and permissions

Ethical approval for study 1 and study 2 was granted by the Science, Technology, Engineering, and Mathematics Ethical Review Committee at the University of Birmingham. For study 1, participants provided written consent to participate in the study. Parents of participants were given the opportunity to opt their child out of participation in the study. For Study 2, participants aged 16 years and over provided written consent to participate in the study. Parents of participants aged below 16 years provided written consent for their child to participate in the study.

### Consent for publication

Not applicable.

## Abbreviations

ADOS: Autism Diagnostic Observation Schedule
ASD: autism spectrum disorder
CA: chronological age
CdLS: Cornelia de Lange syndrome
FXS: fragile X syndrome
IQ: intellectual quotient
RTS: Rubinstein-Taybi syndrome
SCAS-P: Spence Child Anxiety Scale—Parent Version
SCQ: Social Communication Questionnaire
SEN: special educational needs
TD: typically developing
VABS: Vineland Adaptive Behavior Scale
